# Evidence of mosquito-transmitted flavivirus circulation in Piedmont, north-western Italy

**DOI:** 10.1186/1756-3305-5-99

**Published:** 2012-05-22

**Authors:** Francesco Cerutti, Mario Giacobini, Andrea Mosca, Ivan Grasso, Luisa Rambozzi, Luca Rossi, Luigi Bertolotti

**Affiliations:** 1 Department of Animal Production, Epidemiology and Ecology, University of Torino, Torino, Italy; 2 Molecular Biotechnology Center (MBC), University of Torino, Torino, Italy; 3 Istituto per le Piante da Legno e l’Ambiente (IPLA), Regional Government-owned Corporation of Regione Piemonte, Torino, Italy; 4 Current address: Department of Animal Production, Epidemiology and Ecology, University of Torino, Italy, Via Leonardo da Vinci, 44, 10095, Grugliasco, Torino, Italy

**Keywords:** Usutu virus, Insect Flavivirus, *Culex pipiens*, *Ochlerotatus caspius*

## Abstract

**Background:**

*Flavivirus* is a highly heterogeneous viral genus that includes important human pathogens and several viral strains with unknown zoonotic potential. Mosquito-borne flaviviruses have been isolated and characterized in Northern Italy: West Nile virus and Usutu virus were detected in mosquitoes and in different host species and recent studies provided evidence about the circulation of “insect Flavivirus” strains.

**Methods:**

In order to clarify the diffusion and the distribution of the mosquito-transmitted flaviviruses in Italy, we analyzed *Culex* and *Ochlerotatus* mosquitoes collected in 2009 and 2010 in an area divided evenly between hills and plains and where the landscape is dominated by mixed agricultural patches, rice fields, deciduous tree forests, and urban environments. Each mosquito pool was tested for the presence of *Flavivirus* strains and we characterized positive samples by genetic sequencing.

**Results:**

Positive mosquito pools revealed low infection prevalence, but suggested a continuous circulation of both Usutu virus and insect Flavivirus. Interestingly, phylogenetic analyses based on NS5 gene partial sequences showed a closer relationship among new Usutu virus strains from Piedmont and the reference sequences from the Eastern Europe, with respect to Italian samples characterized so far. Moreover, NS5 gene phylogeny suggested that mosquito flaviviruses found in Italy could belong to different lineages.

**Conclusions:**

Our results contribute to a wider point of view on the heterogeneity of viruses infecting mosquitoes suggesting a taxonomical revision of the Mosquito-borne *Flavivirus* group.

## Background

*Flavivirus* is a highly heterogeneous viral genus that includes more than 70 viruses [1], some of which are important human pathogens, including Dengue virus, Japanese encephalitis virus, Yellow fever virus, and West Nile virus (WNV). However, other mosquito-transmitted flaviviruses seem to have a potential pathogenic role in human public health: amongst them, Usutu virus (USUV), isolated in a variety of Central European birds with encephalitis, myocardial degeneration, and necrosis in liver and spleen [2,3], was recently identified in Italy in immunocompromised patients [4,5]. The eco-epidemiology of USUV is still poorly known: Savini and colleagues [6] provided a detailed review about ecological traits of USUV and described the state of the art about the possible epidemiological role of its hosts. They highlighted how improving information on USUV biologic features, distribution host spectrum, and pathogenesis is advisable.

Other flaviviruses, generally named “insect flavivirus”, have so far been isolated only in mosquitoes. *Culex Flavivirus* (CxFV), *Aedes Flavivirus* (AeFV), and Kamiti River virus (KRV), which are able to replicate *in vitro* only in mosquito cells but not in mammalian cells [7,8].

In recent years, several novel flaviviruses were discovered and characterized all over the world. Recently in Spain, Vasquez and colleagues [9] characterized 3 different strains isolated from mosquitoes, identifying two novel insect flaviviruses. Their results seem to suggest that each insect flavivirus is maintained in a specific host genus. The same relation between viral strain and mosquito hosts seems to be present in the Emilia-Romagna region in Northern Italy, where insect flaviviruses were detected in field-collected mosquitoes within an arboviral survey conducted in 2007 and 2008 [10]. The partial NS5 gene sequences suggested a strict relationship between viral strains and mosquito species, as depicted by the phylogenetic tree reported by the authors, where sequences from *Ochlerotatus caspiusAedes albopictus* and *A. vexans* associate in three distinct clades.

In order to increase the knowledge on the diffusion and the genetic heterogeneity of flaviviruses in Italy, we collected mosquitoes in the Eastern part of the Piedmont region, North-Western Italy, and analyzed them for viral presence by using molecular techniques. A recent investigation on the ecology of mosquito populations in the same study area showed that the environmental features can support the maintenance of competent vectors for mosquito-borne diseases [11]. In this framework it is important to identify and characterize potentially zoonotic agents in the study areas, in order to better clarify the diffusion of mosquito-borne diseases in Northern Italy.

## Methods

### Study area and mosquito collection

Our study area includes 65 municipalities located in the Eastern part of Piedmont (population: 120,593 inhabitants, total area: 987 km^2^, centroid: 45.074837° N, 8.392713° E), in North-Western Italy. Hills (mean elevation 268 m) and plains are present in similar proportion, and the landscape is characterized by mixed agricultural patches interspersed with rice fields, deciduous forests and urban areas. Mosquitoes were collected in the frame of a regional project for mosquito control, carried out by IPLA (Istituto per le Piante da Legno e l'Ambiente). A total of 36 CO_2_-baited traps were placed weekly from May to mid September in 2009 and 2010. In order to increase the probability of virus detection, based on literature [10,12], mosquitoes collected in all the 36 traps in one late Summer session of each year underwent the molecular analyses. All mosquitoes were identified [13], grouped by species, collection date and site, and finally pooled with average pool size of 20 individuals. Mosquito pools were placed in 2 mL centrifuge tubes and stored at −80°C until processed for RNA extraction.

### RNA extraction and PCRs

Pools of mosquitoes were re-suspended in 650 μL of RLT buffer (Qiagen, Hilden, Germany) +10% β-mercaptoethanol (Sigma-Aldrich), and homogenized using the Qiagen Tissuelyser (Qiagen, Hilden, Germany) with three 2 mm-metal beads. After a brief centrifugation, mosquito residuals were removed and the supernatant was processed according to the Qiagen RNeasy Mini kit protocol (Qiagen, Hilden, Germany). At the final step, RNA was eluted twice in 30 μL of RNA-free water for a final volume of 60 μL. After quantification with Thermo Scientific Nanodrop 2000 (Thermo-Scientific, Euroclone, Milan, Italy), up to 1 μg of RNA underwent reverse-transcription reaction according to manual instructions (Qiagen QuantiTect Reverse Transcription Kit).

The diagnostic PCR amplifies a conserved region of the NS5 gene, as previously reported [14]. A volume of 5 μl of the cDNA was then used for the first PCR, using generic *Flavivirus* primers. We slightly modified the protocol reported in the original paper using 5 U of HotStarTaq DNA Polymerase (Qiagen), 40 pmol of each primer (Flavi1+, Flavi1-), and 10 nmol of each dNTP. In the nested PCR mix, 1 μL of PCR product from the first reaction was added to 49 μL of reaction mix composed by 1.25 U of HotStarTaq DNA Polymerase, 40 pmol of each primer (Flavi2+, Flavi2-), and dNTPs 10 nmol each dNTP. Products of the nested PCR were analyzed by electrophoresis with a 2% agarose gel (Sigma-Aldrich) and visualized by staining with 0.1% of ethidium bromide.

In order to better characterize Usutu viral strains circulating in the study area, a partial region within the envelope gene was obtained by the amplification of 2 overlapping fragments, according to previous work [15].

### Sequencing

A first DNA sequencing was performed to identify the virus using the final product of the diagnostic nested PCR (130 bp). DNA was purified (NucleoSpin® Extract II, Macherey-Nagel GmbH & Co. KG, Düren, Germany) and quantified before sequencing. All the sequence reactions were performed by BMR Genomics srl (Padua, Italy). The 262 bp amplicon was obtained using Flavi_2_F as forward primer, and cFD2 as reverse, according to the protocol reported by Kuno [16]. In the case of double peaks along the chromatogram, PCR products were purified and cloned, according to the TOPO® XL PCR Cloning Kit (Invitrogen, Carlsbad, CA). Briefly, after the cloning reaction and transformation of chemically competent *E. coli*, selecting culture was performed at 37°C overnight on LB medium (Sigma-Aldrich) with 50 μg/mL kanamycin (Sigma-Aldrich), according to the manufacturer’s instructions. Colony PCR was then performed to analyze positive transformants using the universal primers M13 [17]. Colonies whose PCR product was of the expected size were then expanded in 2 ml LB at 37°C overnight. After plasmid purification (QIAprep Spin Miniprep Kit, Qiagen), inserts were directly sequenced using the M13 primers.

### Phylogenetic and statistical analyses

Sequences were assembled and hand-edited using the computer programs 4Peaks Version 1.7.2 (Mekentosj Inc, Amsterdam, The Netherlands) and Mesquite v. 2.72 [18]. Then, they were aligned with respect to published *Flavivirus* homologous sequences using ClustalW [19], with manual adjustment. Phylogenetic analyses were conducted using Bayesian methods implemented in the software MrBayes v. 3.2 [20,21], with a following taxon ordering based on the uncorrected nucleotide diversity among samples, according to [22]. Nucleotide and protein diversity was evaluated with PAUP* 4.0 [23]. Flavivirus infection prevalence was estimated considering the pooling of samples. The related Minimum Infection Rate (MIR) and Maximum Likelihood Estimation (MLE) were calculated using the PooledInfRate statistical software package [24]. Briefly, the MIR assumes that a positive pool contains only one infected mosquito, while MLE assumes a binomial distribution of positive mosquitoes among pools and calculates the infection rate most likely observed from the results. Both measures are expressed as the number of infected mosquitoes per 1000 tested.

## Results

A total of 4,228 mosquitoes were collected in the 36 traps included in the study area. In late August 2009 (yearly week 35) we processed 1,912 *C. pipiens* (96 pools), 1,009 *O. caspius* (70 pools) and 631 *C. modestus* (18 pools). In 2010 (mid September, week 37), we processed additional 223 *C. pipiens* (12 pools) and 453 *O. caspius* (24 pools).

Among the analyzed mosquitoes, positivity to *Flavivirus* was detected only in pools of *C. pipiens* and *O. caspius*. In particular, two pools of *C. pipiens* and one pool of *O. caspius* were positive to the *Flavivirus* genus in 2009, while in 2010 one *C. pipiens* pool and three *O. caspius* pools were positive. In 2010, all *O. caspius* positive pools were collected in the same location. Molecular characterization of the virus was performed by sequencing the products of the diagnostic nested PCR. All sequences obtained from *C. pipiens* pools were characterized as Usutu virus, while *O. caspius* mosquitoes carried a presumed insect-specific *Flavivirus* related to those published by Calzolari and coauthors [10]. The prevalence of both Usutu and insect *Flavivirus* was comparable to the ones found in other Italian regions [10]. MIRs and MLEs for the inferred prevalence of infection in mosquitoes are reported in Table 1.

**Table 1:** Collection data, MIR, MLE of the mosquitoes collected in the study. MIR and MLE are expressed as the number of infected mosquitoes per 1000 tested; CI 95%: 95% confidence interval; na: not applicable

**Collection date Year (week)**	**Mosquito species**	**Tested specimens**	**Tested pools**	**Positive pools**	**MIR (CI 95%)**	**MLE (CI 95%)**
2009 (35)	*C. pipiens*	1912	96	2	1.05 (0.0-2.49)	1.05 (0.19-3.43)
	*O. caspius*	1009	70	1	0.99 (0.0-2.93)	0.99 (0.06-4.77)
	*C. modestus*	631	18	0	na	na
2010 (37)	*C. pipiens*	223	12	1	4.48 (0.0-13.25)	4.37 (0.26-21,06)
	*O. caspius*	453	24	3	6.62 (0.0-14.09)	6.92 (1.84-18.83)

**Figure 1:**
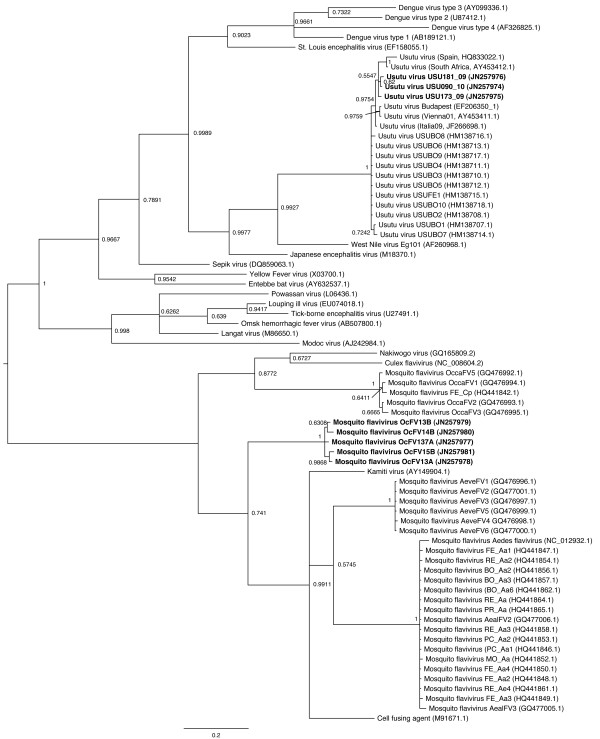
**Phylogenetic tree of Flavivirus genus.** A portion of 226 bp on the NS5 gene was used to infer the tree. Internal nodes are labeled with the posterior probability of the node. The tree was built using MrBayes Software and the following parameters: GTR + G evolutionary model, 3,000,000 generations, sample frequency = 100, burn-in = 3000. Taxa order in the tree is adjusted according to the uncorrected p genetic distance between samples.

A phylogenetic tree was built considering a 226 bp fragment of the NS5 gene of the *Flavivirus* genus, in order to infer the relationships between our samples and the reference sequences available online. As reported in Figure 1, two of the Usutu positive samples were closely related to the strains isolated in Vienna, Austria (AY453411) and Budapest, Hungary (EF206350) and less related to the isolates collected in Italy.

In the same tree, the positive samples from *O. caspius* created a new insect flavivirus clade, divergent by the insect flaviviruses found by Calzolari and coauthors [10]. Genetic distance analysis between all the Italian and Spanish insect flavivirus sequences showed a similarity of 72.18% and 65.99% comparing new obtained sequences to Mediterranean Flaviviruses from *O. caspius* and *C. pipiens* respectively [9]. These data suggest that the viral strain might represent a new divergent strain among insect flaviviruses.

Usutu *env* sequences obtained in this study confirmed the relationship of new Italian strains with the isolates from Austria and Hungary. In detail, a very low degree of heterogeneity has been found among the new Italian samples (mean nucleotide similarity = 99.65%, mean amino acid similarity = 100%), in agreement with the work conducted by Chvala and colleagues [25]. Moreover, we detected only non-synonymous substitutions comparing our new sequences with the closest reference strains Vienna 2001 [26] and Budapest [2], confirming a high similarity among the European samples (Table 2).

**Table 2:** **Nucleotide substitutions in the*env*partial sequence among new Italian USUV strains and reference sequences**

**USUV strains**	**Genbank accession number**	**Nucleotide position**^**a**^																			
		**1209**	**1242**	**1251**	**1294**	**1335**	**1344**	**1351**	**1458**	**1470**	**1554**	**1731**	**1771**	**1785**	**2073**	**2076**	**2121**	**2181**	**2196**	**2463**	**2466**
Vienna2001	AY453411	g	g	c	c	c	g	**a**	a	g	a	t	t	t	c	c	c	c	c	a	c
Budapest	EF206350	.	.	.	.	.	.	**.**	.	.	.	.	.	.	.	.	.	t	.	.	.
USU629_05	EF393681	.	.	.	.	.	.	**.**	g	a	.	.	.	.	.	.	.	.	.	.	.
USU502_03	EF078297	.	.	.	.	.	.	**.**	.	.	.	.	.	.	.	.	.	.	.	.	.
USU281_03	EF078294	.	.	.	.	.	.	**.**	.	.	.	.	.	.	.	.	.	.	.	.	.
USU623_04	EF078300	.	.	.	.	t	.	**.**	.	.	.	.	.	.	.	.	.	.	.	.	.
USU618_04	EF078299	.	.	.	.	.	.	**.**	.	.	g	.	.	.	.	.	.	.	.	.	.
USU499_03	EF078296	.	.	.	.	.	.	**.**	.	.	.	.	.	.	t	.	.	.	.	.	.
USU450_03	EF078295	.	a	.	.	.	.	**.**	.	.	.	.	.	.	.	.	.	.	.	.	.
USU604_05	EF393680	a	.	t	.	.	.	**g**	.	.	.	c	.	.	.	.	.	.	.	.	.
USU588_05	EF393679	a	.	t	.	.	.	**g**	.	.	.	c	.	.	.	.	.	.	.	.	.
USU589_05	EF078301	a	.	t	.	.	.	**g**	.	.	.	c	.	.	.	.	.	.	.	.	.
USU338_04	EF078298	.	.	.	.	.	.	**.**	.	.	.	.	c	c	.	.	t	.	.	.	t
USU090_10	JN257974	.	.	.	t	.	a	**.**	.	a	.	.	.	.	.	.	.	.	.	g	.
USU181_09	JN257976	.	.	.	.	.	.	**.**	.	.	.	.	.	.	.	t	.	.	.	g	.
USU173_09	JN257975	.	.	.	.	.	.	**.**	.	.	g	.	.	.	.	.	.	.	t	.	.

Dots indicate identity respect to Vienna2001 reference strain. The unique non-synonymous substitution at position 1351 is reported in bold.

^a^ Nucleotide positions refer to the complete genome sequence of USUV Vienna 2001 (AY453411).

All newly generated sequences have been deposited into GenBank (accession numbers: USUV partial NS5 gene JN257974-6, insect Flavivirus partial NS5 gene JN257977-81, USUV partial *env* gene JN257982-4).

## Discussion

In Northern Italy, West Nile and Usutu viruses were detected and they co-circulate in areas characterized by high abundance of arthropod vectors [12]. Interestingly, a recent study demonstrated enhanced transmission of WNV by *C. quinquefasciatus* mosquitoes simultaneously infected with *Culex Flavivirus* under laboratory conditions [27]. In addition, the high levels of genetic heterogeneity showed by Flaviviruses may point towards a greater variability of the host species. In this framework, it is particularly important to identify and characterize circulating viral strains within a defined area, in order to understand epidemiological main rules that can drive pathogen diffusion and distribution.

In this study, we collected mosquitoes in the same study area during the late summer of two consecutive years, and we molecularly tested the mosquitoes to investigate flavivirus circulation.

Positive mosquito pools suggested a low infection prevalence and a continuous circulation of both USUV and insect flaviviruses. Interestingly, phylogenetic analyses based on NS5 gene partial sequences showed a closer relationship among new USUV strains from Piedmont and the reference sequences from the Eastern Europe, compared with other Italian isolates characterized by Calzolari and colleagues [12]. A recent work presented new sequences of Usutu virus isolated from blackbirds, human cerebrospinal fluid and *C. pipiens* mosquitoes [6] and compared them with available data. All the sequences from mammalians clustered together in a separated clade, while the sample extracted by mosquito pools clustered with the Austrian and Hungarian clades. Possibly due to the small number of homologue sequences, the analyses conducted on the partial region of the *env* gene do not support the hypothesis of segregation by host. However, it seems that a clear relationship exists among the new USUV strains characterized in Piedmont and the strains isolated in Austria and Hungary. This point is particularly intriguing, as the role of migratory birds in USUV ecology is still poorly understood. For this reason, the investigation of USUV in avian species, considering both migratory and resident birds, will be particularly important. In the same manner, the correct characterization of circulating strains, by means of longer genetic sequences, is essential for the right strain identification and to understand the evolutionary features of this virus in vectors and hosts.

The phylogenetic inference on the 226 bp fragment of NS5 gene of the genus *Flavivirus* suggests that mosquito flaviviruses found in Italy could belong to different species. Most of the insect flaviviruses found by Calzolari and colleagues in *A. albopictus* cluster with the *Aedes Flavivirus*[10], but many others seem to belong to different clades within the *Flavivirus* genus, closely related to *Culex Flavivirus*[28] and the similar Cell fusing agent virus [29]. The new flavivirus sequences detected in this study formed a divergent clade in the phylogenetic tree increasing the genetic heterogeneity within the insect flavivirus group. Though only 226 bp long sequences, those are clearly separated from the sequences obtained from *O. caspius* mosquitoes in Emilia Romagna region (closer to Culicinae *flavivirus* PoMoFlav), suggesting that different mosquito species can be infected by different strains.

## Conclusions

Our results contribute to a wider point of view on the heterogeneity of viruses infecting mosquitoes. In fact, we should consider the ecology of mosquito-borne flaviviruses as more complex than expected. Many known and unknown viruses may play an important role in infecting different hosts, interacting with each other, and promoting or reducing the host fitness toward new infections.

Further investigations and deeper genetic characterizations are needed, in order to clarify new viral strain relationships among mosquito-borne viruses as well as their presence in different host populations. Finally, given the extremely large genetic heterogeneity found among insect flaviviruses, a taxonomical revision of this viral group, considering genetic peculiarities, *in vitro* properties and vector/host interactions, seems necessary.

In conclusion, our results confirmed that potentially zoonotic flaviviruses are circulating also in North-Western Italy as the circulation of zoonotic WN and USUTU virus is very well known in North-Eastern Italy since 2008 and many papers and official reports have been published. In this scenario, it will be important to characterize the ecological features constituting the natural life cycle of these viruses and understand the correct relationship between virus strains, vector and host species.

## Competing interests

The authors declare that they have no competing interests.

## Authors' contributions

FC, MG, AM, LRambozzi, LRossi and LB participated in the conceptualization of the study. AM and IG coordinated mosquitoes collection and identification. FC and LB carried out the molecular and phylogenetic analyses. FC, MG and LB drafted the manuscript. All authors participated in the revision of the manuscript and approved the submitted version. All authors read and approved the final manuscript.
